# Clinical Features and Outcomes of Spontaneous Tumor Lysis in Testicular Germ Cell Tumors: A Case Series From a Cancer Center in Lahore, Pakistan

**DOI:** 10.7759/cureus.104882

**Published:** 2026-03-09

**Authors:** Lokesh Kumar, Nahel Chaudhry, Syed Abdullah Javaid Bukhari

**Affiliations:** 1 Internal Medicine, Shaukat Khanum Memorial Cancer Hospital and Research Centre, Lahore, PAK; 2 Medical Oncology, Shaukat Khanum Memorial Cancer Hospital and Research Centre, Lahore, PAK

**Keywords:** acute spontaneous tumor lysis syndrome, carboplatin, platinum chemotherapy, rasburicase, renal replacement therapy (rrt), testicular germ cell tumor

## Abstract

Background

Testicular germ cell tumors (TGCTs) are the most common solid malignancies in young adult men and typically have excellent outcomes with modern therapy. Tumor lysis syndrome (TLS) is a rare but potentially fatal complication in TGCTs, particularly when it occurs spontaneously in the absence of cytotoxic treatment. Data describing spontaneous TLS in this setting remain limited.

Methods

We report a case series of six patients who presented with spontaneous TLS secondary to TGCTs. Clinical features, imaging findings, laboratory results, management strategies, and outcomes were reviewed retrospectively from the electronic medical records. TLS was diagnosed based on characteristic biochemical abnormalities, including hyperuricemia, hyperphosphatemia, hyperkalemia, hypocalcemia, and acute kidney injury (AKI).

Results

Most patients had seminomatous histology, and all demonstrated extensive tumor burden on imaging. TLS was identified prior to the initiation of chemotherapy in all cases. Management included aggressive intravenous hydration, urate-lowering therapy with rasburicase and/or allopurinol, and renal replacement therapy for refractory metabolic disturbances. Chemotherapy was initiated with individualized dosing strategies to minimize further metabolic complications. Despite early recognition and multidisciplinary management, mortality was substantial, largely due to renal failure and infectious complications.

Conclusions

Spontaneous TLS is a rare but life-threatening complication of TGCTs, particularly in patients with bulky disease. Tumor burden appears to be a key risk factor regardless of histological subtype. Increased clinical awareness, early diagnosis, aggressive supportive care, and tailored chemotherapy approaches are critical to reducing morbidity and mortality in this high-risk population.

## Introduction

Testicular germ cell tumors (TGCTs) constitute approximately 1% of male malignancies but are the most common solid organ tumors in males aged 15-35 years, accounting for 95% of malignant testicular neoplasms [1]. Tumor lysis syndrome (TLS) is a potentially fatal oncologic emergency characterized by rapid cellular destruction, resulting in metabolic disturbances including hyperkalemia, hyperphosphatemia, hypocalcemia, hyperuricemia, and consequent acute kidney injury (AKI) [2-5]. TLS commonly follows cytotoxic therapy but can rarely occur spontaneously, particularly in tumors with high proliferation rates and tumor burden, such as Burkitt lymphoma, inflammatory breast cancer, and metastatic germ cell tumors [4,6]. Pathophysiology involves the massive release of intracellular potassium, phosphate, and nucleic acids, leading to electrolyte imbalances and uric acid nephropathy [4,7-9]. The resultant metabolic derangements may precipitate cardiac arrhythmias, seizures, systemic inflammatory response syndrome (SIRS), and multiorgan failure [3].

Spontaneous TLS in TGCTs is rare, with limited cases reported. We present a series of six patients with spontaneous TLS secondary to TGCTs, detailing clinical features, biochemical profiles, management approaches, and outcomes.

## Materials and methods

This study is a retrospective case series of patients diagnosed with spontaneous tumor lysis syndrome (TLS) in the setting of testicular germ cell tumors (TGCTs). Six patients presenting to the tertiary oncology center were identified from January 2022 to December 2024. Inclusion criteria comprised a confirmed diagnosis of TGCT based on histopathology or tumor markers and evidence of spontaneous TLS occurring prior to the initiation of cytotoxic chemotherapy or radiotherapy. TLS was confirmed using the Cairo-Bishop classification, and all the patients had clinical TLS. The Institutional Review Board (IRB) of Shaukat Khanum Memorial Cancer Hospital and Research Centre, Lahore, issued approval EX-02-05-25-03.

Clinical data were extracted from the electronic medical records and included patient demographics, presenting symptoms, tumor histology, the extent of disease on imaging, and baseline comorbidities. Radiological assessment with computed tomography (CT) was reviewed to determine tumor burden, including primary tumor size and the presence of metastatic disease.

Laboratory parameters were collected at presentation and during hospitalization, including serum uric acid, potassium, phosphorus, calcium, creatinine, and lactate dehydrogenase (LDH) levels. TLS was defined according to standard laboratory and clinical criteria, characterized by hyperuricemia, hyperphosphatemia, hyperkalemia, hypocalcemia, and acute kidney injury in the absence of recent anticancer therapy.

Management strategies were documented, including intravenous fluid administration, the use of urate-lowering agents (rasburicase and/or allopurinol), electrolyte correction, the need for renal replacement therapy, and the timing and modification of chemotherapy initiation. Clinical outcomes assessed included the resolution of metabolic abnormalities, the development of complications, the need for intensive care support, and in-hospital mortality.

Given the descriptive nature and small sample size of this case series, data were analyzed using descriptions.

## Results

Case 1

**Figure 1:**
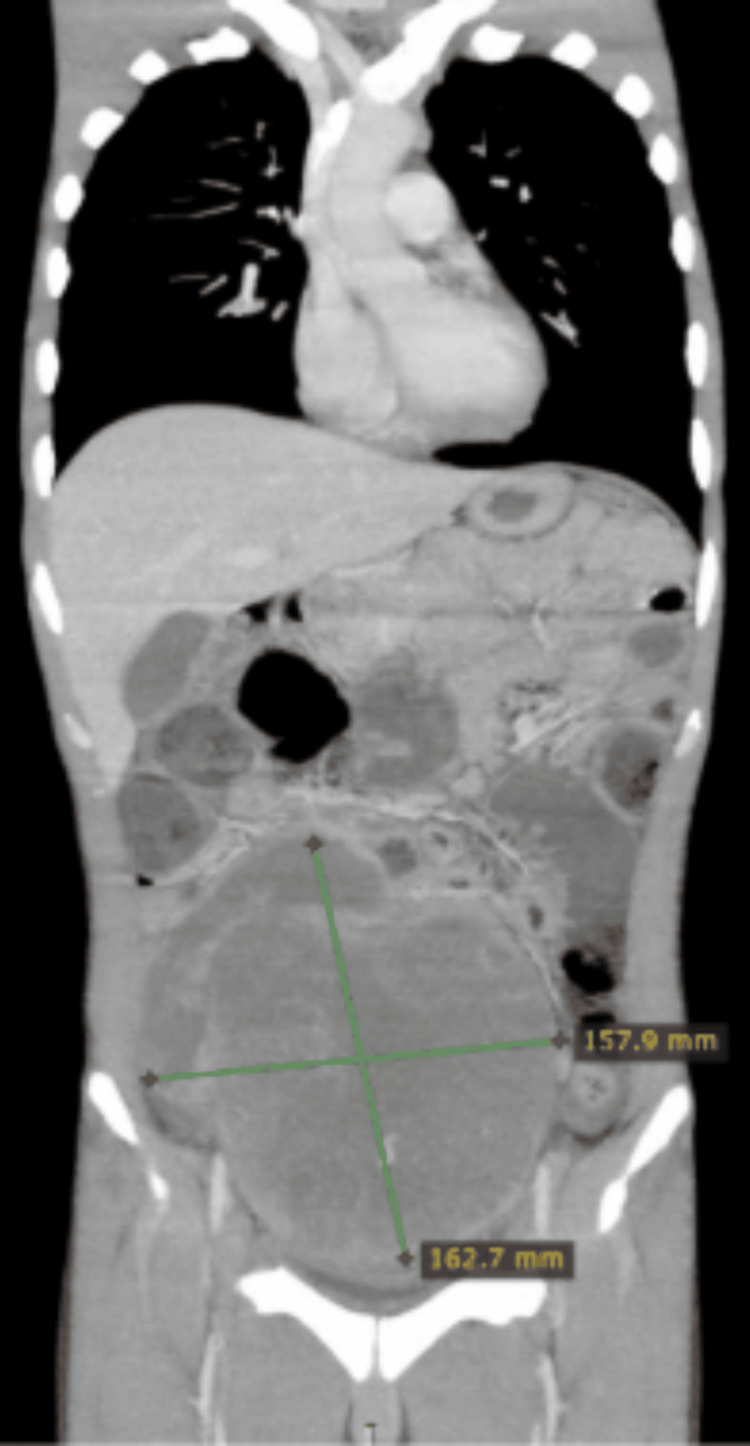
Coronal plane of the CT scan showing a mass of around 11×15 cm CT: computed tomography

**Figure 2:**
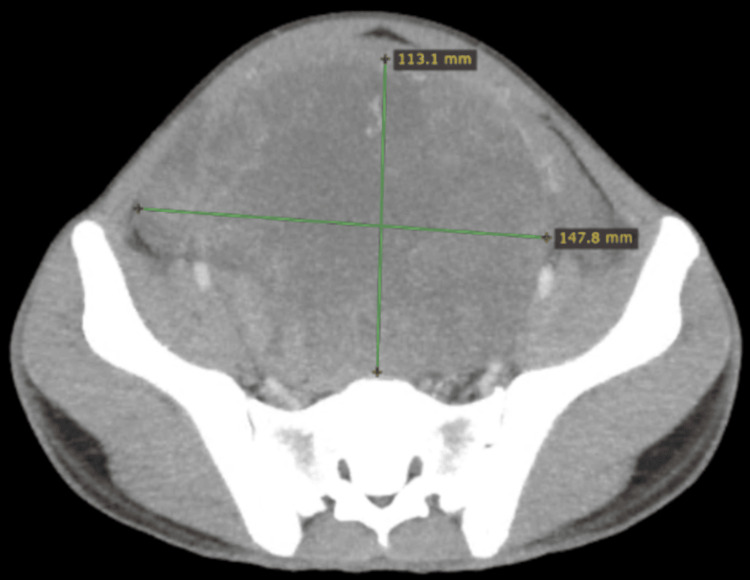
Axial planes of the CT scan showing a mass of around 11×15 cm CT: computed tomography

A 21-year-old man presented with a two-month history of lower abdominal pain and unintentional weight loss. Physical examination revealed an empty scrotum and a palpable lower abdominal mass extending up to the level of the umbilicus. Ultrasound of the abdomen and scrotum identified a solid-cystic mass in the lower abdomen consistent with undescended testes. Subsequent contrast-enhanced computed tomography (CT) of the chest, abdomen, and pelvis (CAP) demonstrated a 15 cm abdominopelvic mass without evidence of pelvic lymphadenopathy (Figures 1, 2).

The patient was admitted from the outpatient oncology clinic for the initiation of inpatient chemotherapy due to his poor general condition. Baseline laboratory investigations prior to chemotherapy revealed biochemical abnormalities consistent with spontaneous tumor lysis syndrome (Table 1). The patient was managed with aggressive intravenous hydration and a single 6 mg dose of rasburicase; however, metabolic parameters failed to improve significantly, necessitating the initiation of hemodialysis. Following one session of dialysis, renal function improved substantially.

**Table 1:** Blood results pre- and post-rasburicase, pre- and post-hydration, and after renal replacement AFP, alpha-fetoprotein; Cr, creatinine; K, potassium; Pho, phosphate; LDH, lactate dehydrogenase; HCG, human chorionic gonadotropin; HDx, hemodialysis

	Reference range	Pre-chemo	Post-rasburicase	Post-HDx	Day 7 of rasburicase
AFP	<5.0 IU/mL	>30000	-	-	-
Cr	0.7-1.2	6.06	6.45	3.7	1.54
K	3.5-5.1 mmol/L	5.46	5.15	3.98	3.85
Calcium corrected	8.5-10.5 mg/dL	9.8	9.57	9.68	9.51
Pho	2.5-4.5 mg/dL	5.37	6.8	5.3	3.45
Uric acid	3.4-7.0 mg/dL	10.13	9.6	2.53	2.67
LDH	135-225 U/L	1229	-	1300	-
Beta-HCG	<2 ng/mL	28031	-	-	-

Chemotherapy was commenced using a renal function-adjusted bleomycin, etoposide, and cisplatin (BEP) regimen, consisting of bleomycin with 100% dose reduction, etoposide at 50% dose reduction, and carboplatin dosed at area under the curve (AUC) of 5×25 mg (total 125 mg). Supportive hydration was continued alongside the close monitoring of tumor lysis parameters. The patient tolerated the first cycle well and was subsequently discharged. He completed the remaining chemotherapy cycles with a full-dose standard BEP regimen without significant toxicity.

A follow-up CT scan performed four weeks after the completion of chemotherapy demonstrated a partial treatment response. The patient subsequently underwent laparotomy with the excision of the residual abdominal mass and bilateral orchidectomy. Histopathological analysis revealed no residual viable malignancy (right testis, normal morphology, and left testis showed hemorrhage and infarction). He remains under regular surveillance with three-monthly clinical and radiological assessments and has shown no evidence of disease recurrence over 2.5 years of follow-up.

Case 2

**Figure 3:**
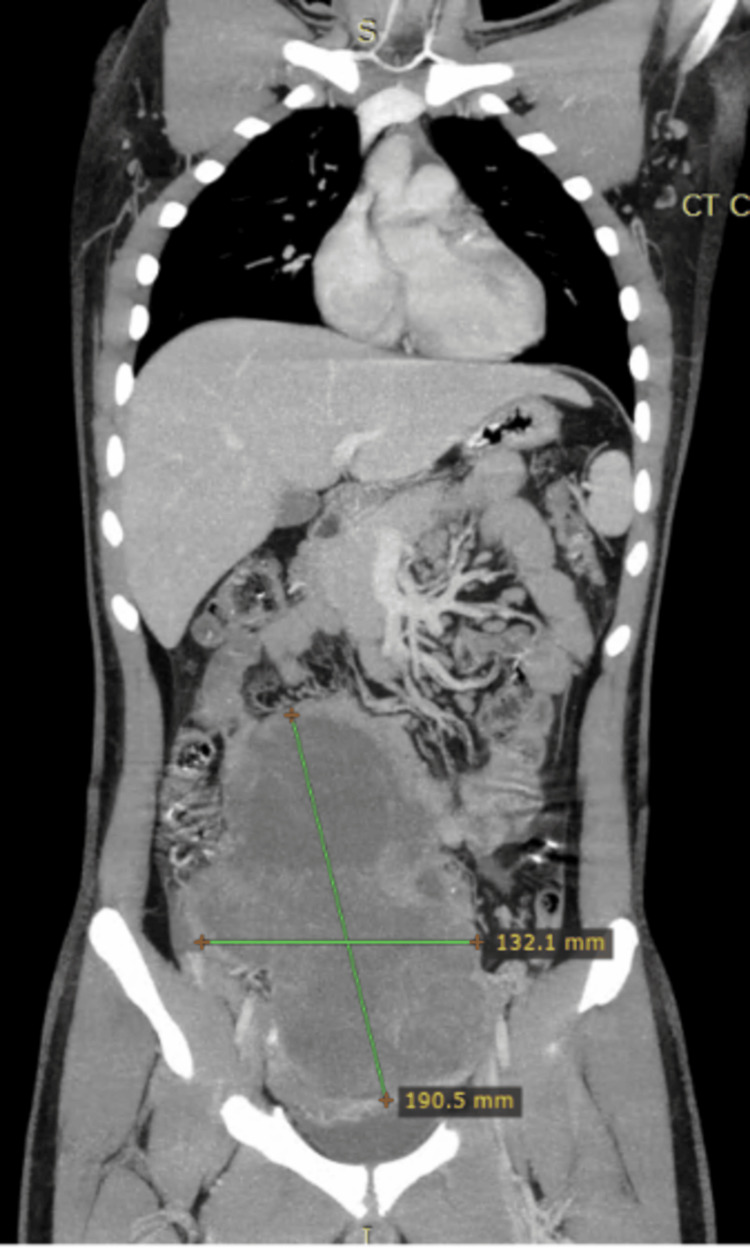
Coronal image of the CT of the abdomen and pelvis showing a mass of around 19×13 cm CT: computed tomography

A 22-year-old man with no significant past medical history presented with a one-month history of abdominal pain. Physical examination revealed a soft abdomen and an underdeveloped, empty scrotum. The ultrasound imaging of the abdomen identified a lower abdominal mass extending into the left inguinal canal, with the absence of the testes within the scrotum. Contrast-enhanced CT scan of the chest, abdomen, and pelvis revealed a 19 cm abdominopelvic mass, accompanied by retroperitoneal lymphadenopathy (Figure 3). Laboratory investigations showed markedly elevated alpha-fetoprotein (AFP; >30000 IU/mL), lactate dehydrogenase (LDH; 565 U/L), and beta-human chorionic gonadotropin (beta-HCG; <2 ng/mL). The histopathological examination of the mass biopsy confirmed a yolk sac tumor.

The patient was admitted for inpatient chemotherapy. Pre-treatment laboratory findings were consistent with tumor lysis syndrome (Table 2). Management included intravenous hydration and a single 6 mg dose of rasburicase, resulting in partial improvement in tumor lysis parameters within 24 hours. Subsequently, chemotherapy was initiated with a modified BEP regimen; etoposide and cisplatin were reduced by 20%, and bleomycin was omitted completely.

**Table 2:** Investigations pre- and post-rasburicase and pre- and post-hydration AFP, alpha-fetoprotein; Cr, creatinine; K, potassium; Pho, phosphate; LDH, lactate dehydrogenase; HCG, human chorionic gonadotropin

	Reference range	Pre-chemo	Post-rasburicase	One week after rasburicase
AFP	<5.0 IU/mL	>30000	-	-
Cr	0.7-1.2	1.75	1.33	1.42
K	3.5-5.1 mmol/L	5.57	5.75	3.59
Calcium	8.5-10.5 mg/dL	9.67	9.37	9.1
Pho	2.5-4.5 mg/dL	7.23	5.17	3.51
Uric acid	3.4-7.0 mg/dL	16.59	11.5	3.67
LDH	135-225 U/L	565	-	-
Beta-HCG	<2 ng/mL	<2	-	-

On the day following chemotherapy initiation, the patient developed severe metabolic acidosis (pH: 7.17) and respiratory distress necessitating invasive mechanical ventilation. Persistent hyperkalemia and progressive renal impairment required the initiation of intermittent hemodialysis. Chemotherapy was continued with cisplatin at 20% dose reduction, while etoposide and bleomycin remained withheld. Supportive care included diuretic therapy and broad-spectrum antibiotics.

Microbiological cultures from tracheal aspirate and blood grew methicillin-sensitive *Staphylococcus aureus*; antimicrobial therapy was adjusted accordingly. Despite aggressive supportive measures, the patient’s clinical condition deteriorated, culminating in death from renal failure/sepsis on hospital day 11.

Case 3

A 40-year-old man presented with abdominal pain and exertional dyspnea. On examination, he was afebrile, emaciated, and dehydrated, with a palpable firm abdominal mass extending into the scrotum. Scrotal ultrasonography revealed the complete replacement of the right testis by a large heterogeneous mass. A tru-cut biopsy of the mass was performed, and histopathological analysis confirmed a diagnosis of yolk sac tumor.

Laboratory investigations on admission demonstrated elevated tumor markers alongside deranged renal function and electrolyte abnormalities (Table 3). Contrast-enhanced computed tomography of the chest, abdomen, and pelvis (CT CAP) showed a right testicular mass with extensive inguinal and intra-abdominal lymphadenopathy, involving regions above and below the diaphragm. Additionally, imaging revealed a right-sided hydronephroureter, necessitating percutaneous nephrostomy placement for urinary decompression.

**Table 3:** Investigations pre- and post-rasburicase and pre- and post-hydration AFP, alpha-fetoprotein; LDH, lactate dehydrogenase; HCG, human chorionic gonadotropin; Ca, calcium

	Reference range	On admission	Post-rasburicase	One week post-rasburicase
Creatinine	0.90-1.30	5.79	4.89	4.03
LDH	135-225 U/L	3167	-	-
Beta-HCG	<2 ng/mL	422.5	-	-
AFP	<2 IU/L	12.7	-	-
Potassium	3.5-5.1 mmol/dL	5.64	5.35	5.92
Uric acid	3.7-7.7 mg/dL	13.5	0.75	<0.5
Phosphorous	2.9-4.7 mg/dL	11.7	10.9	9.19
Ca corrected	8.5-10.5 mg/dL	8.73	9.01	7.2

The patient was diagnosed with spontaneous tumor lysis syndrome based on laboratory findings. He was commenced on aggressive intravenous hydration, along with rasburicase 6 mg and allopurinol, to manage hyperuricemia. Despite these measures, serum creatinine levels remained elevated, necessitating the initiation of intermittent hemodialysis, which was performed daily for three consecutive days. Unfortunately, the patient’s renal function did not improve sufficiently to allow the initiation of chemotherapy. On hospital day 8, he experienced an abrupt decline in neurological status with a Glasgow Coma Scale score dropping to 3/15, followed by cardiopulmonary arrest. Resuscitation efforts were unsuccessful, and the patient expired.

Case 4

A 26-year-old man with no known comorbidities presented with a three-month history of progressive abdominal distension, weight loss, and alternating diarrhea and constipation. The patient had previously undergone an inguinal orchidectomy for undescended testes at a local hospital 1.5 years prior; however, histopathology reports were unavailable, and he did not receive any further treatment or follow-up.

**Figure 4:**
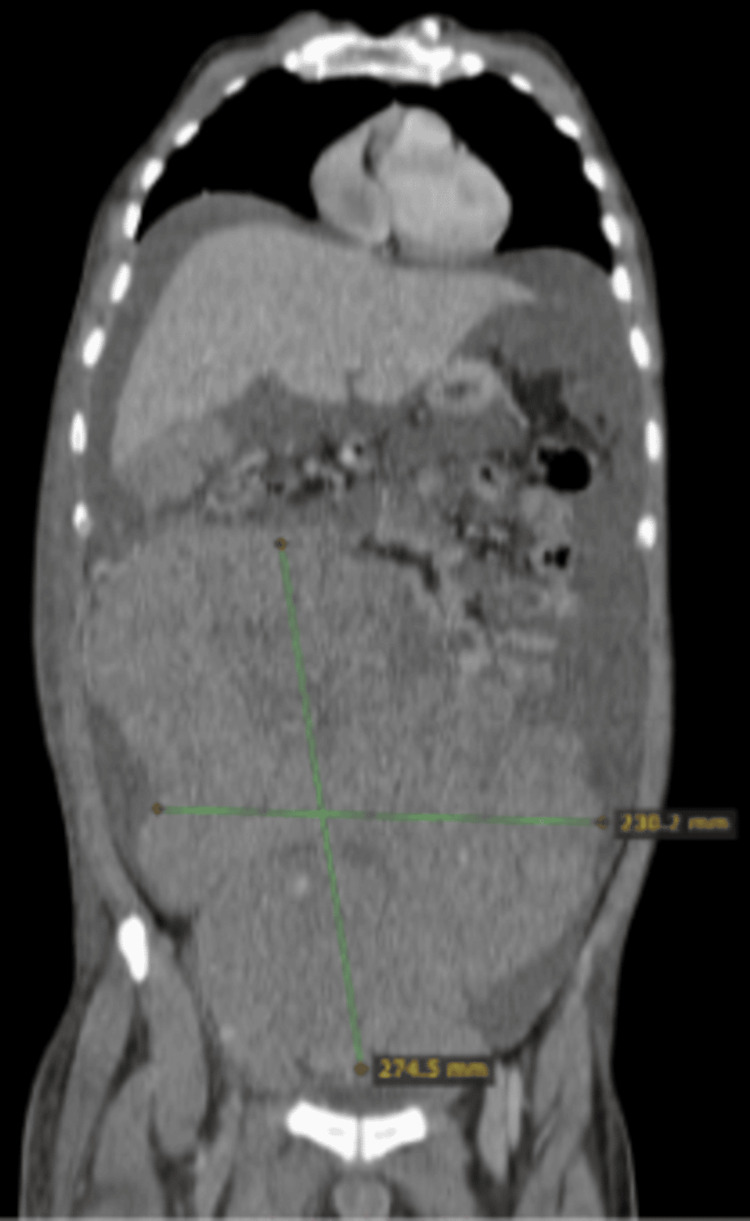
Coronal image of a CT scan showing around 23×17 cm mass in the pelvis CT: computed tomography

**Figure 5:**
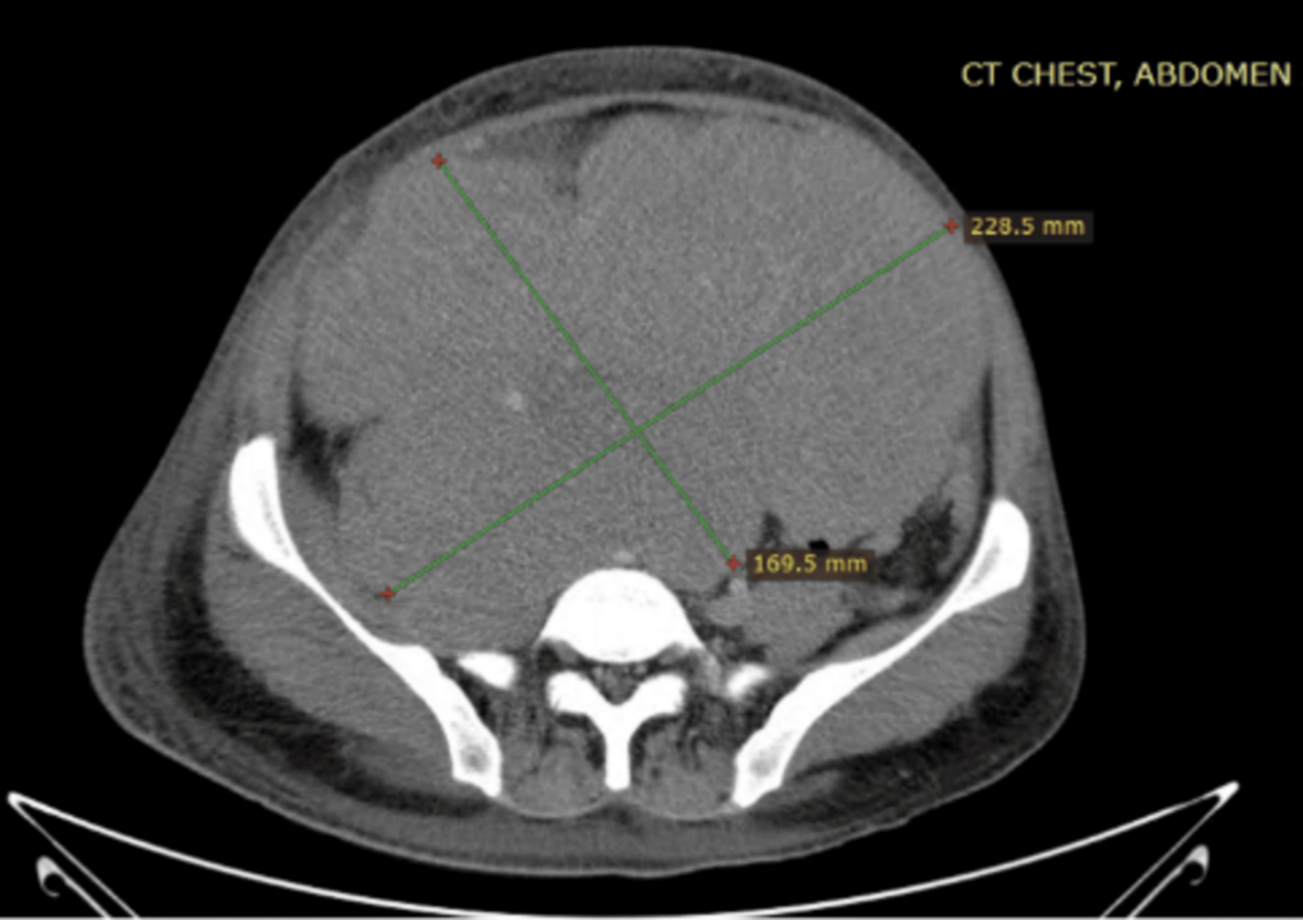
Axial image of a CT scan showing around 23×17 cm mass in the pelvis CT: computed tomography

Baseline laboratory investigations revealed elevated lactate dehydrogenase (LDH) and beta-human chorionic gonadotropin (beta-HCG) levels, while alpha-fetoprotein (AFP), complete blood count, and renal and liver function tests were within normal limits. CT CAP demonstrated a 23 cm centrally located mesenteric abdominal mass, moderate ascites, bilateral para-aortic lymphadenopathy, and mild bilateral hydronephrosis (Figures 4, 5). The histopathological examination of a tru-cut biopsy from the abdominal mass confirmed seminoma.

The patient was diagnosed with obstructive uropathy and underwent bilateral ureteric stenting; however, serum creatinine levels failed to improve. Subsequent investigations indicated ongoing spontaneous tumor lysis syndrome. He was therefore treated with rasburicase and initiated on hemodialysis (Table 4).

**Table 4:** Investigation pre- and post-rasburicase and renal replacement therapy AFP, alpha-fetoprotein; K, potassium; LDH, lactate dehydrogenase; HCG, human chorionic gonadotropin; PO_4_, phosphate

	Reference range	Bloods on presentation	On admission (28/04/22)	Post-dialysis and post-rasburicase	One week post-rasburicase
Uric acid	3.7-7.7 mg/dL	-	31.3	15	6.33
K	3.5-5.1 mmol/dL	4.73	5.7	4.8	4.58
PO_4_	2.9-4.7 mg/dL	-	8.8	5.8	4.79
Calcium	8.5-10.5 mg/dL	-	8.12	8.9	7.54
Creatinine	0.90-1.30	6.2	10.2	5.3	2.01
AFP	<2 IU/L	3.4	-	-	-
LDH	135-225 U/L	6150	-	-	-
Beta-HCG	<2 ng/mL	118	87	-	-

Chemotherapy was commenced with carboplatin (AUC: 5) and etoposide at a 50% dose reduction once his renal functions started to improve. Seven days following the first chemotherapy cycle, renal function normalized. The patient subsequently completed four cycles of full-dose BEP, which he tolerated well. A follow-up CT CAP demonstrated a reduction of the peritoneal mass to 8.9 cm, with the concomitant regression of abdominal lymphadenopathy.

The case was reviewed in a multidisciplinary tumor board meeting, where the residual mass was deemed unresectable. The patient remains progression-free 12 months post-chemotherapy and continues active surveillance.

Case 5

A 35-year-old man presented with a one-year history of swelling in the inguinal region associated with pain, progressive abdominal distension, and weight loss. The patient had a prior admission at another hospital, during which pleural and peritoneal fluid drainage was performed, and two sessions of dialysis were reportedly administered; however, no medical records were available for review.

**Figure 6:**
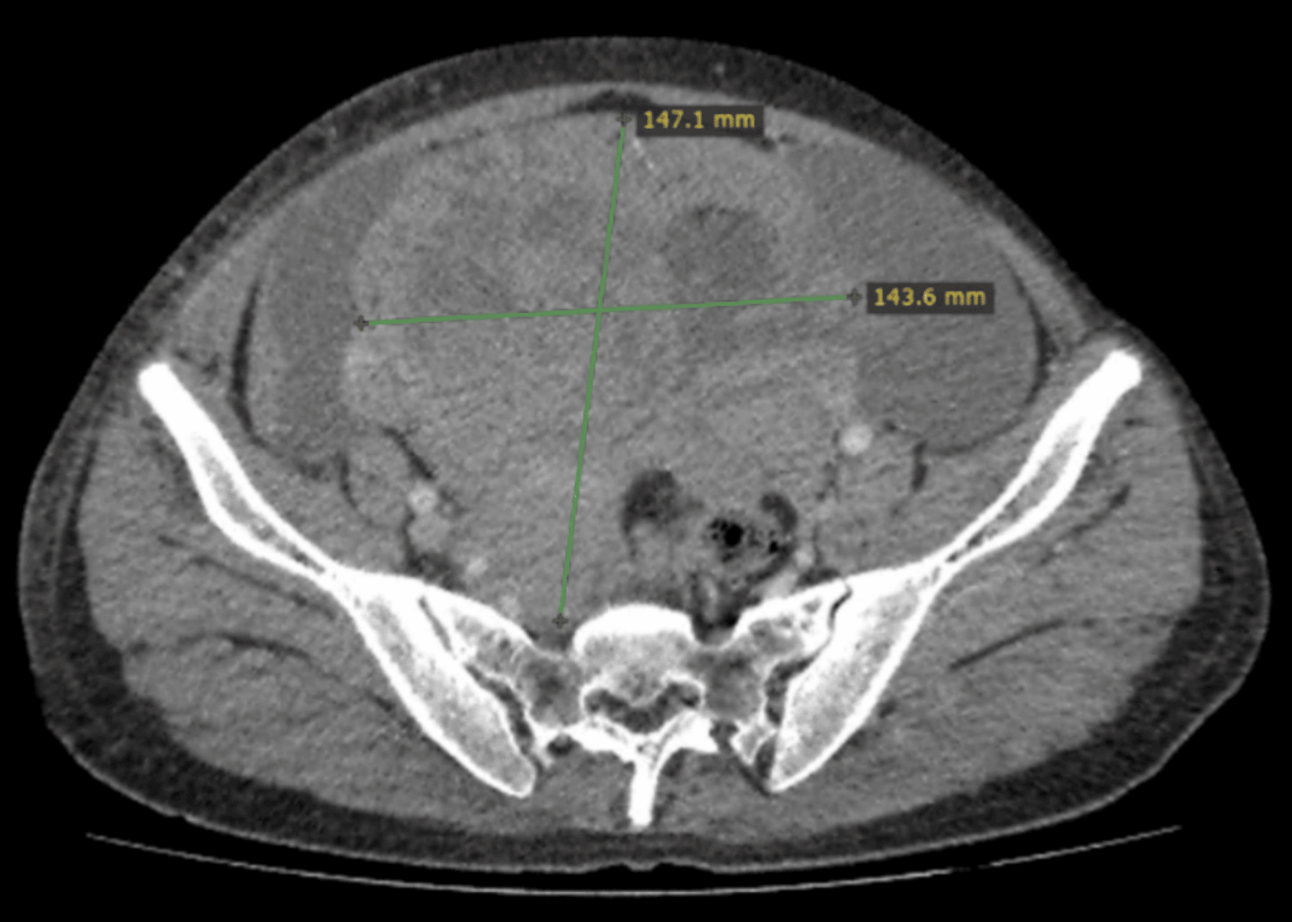
Axial image of a CT scan showing around 15×15 cm mass in the pelvis CT: computed tomography

**Figure 7:**
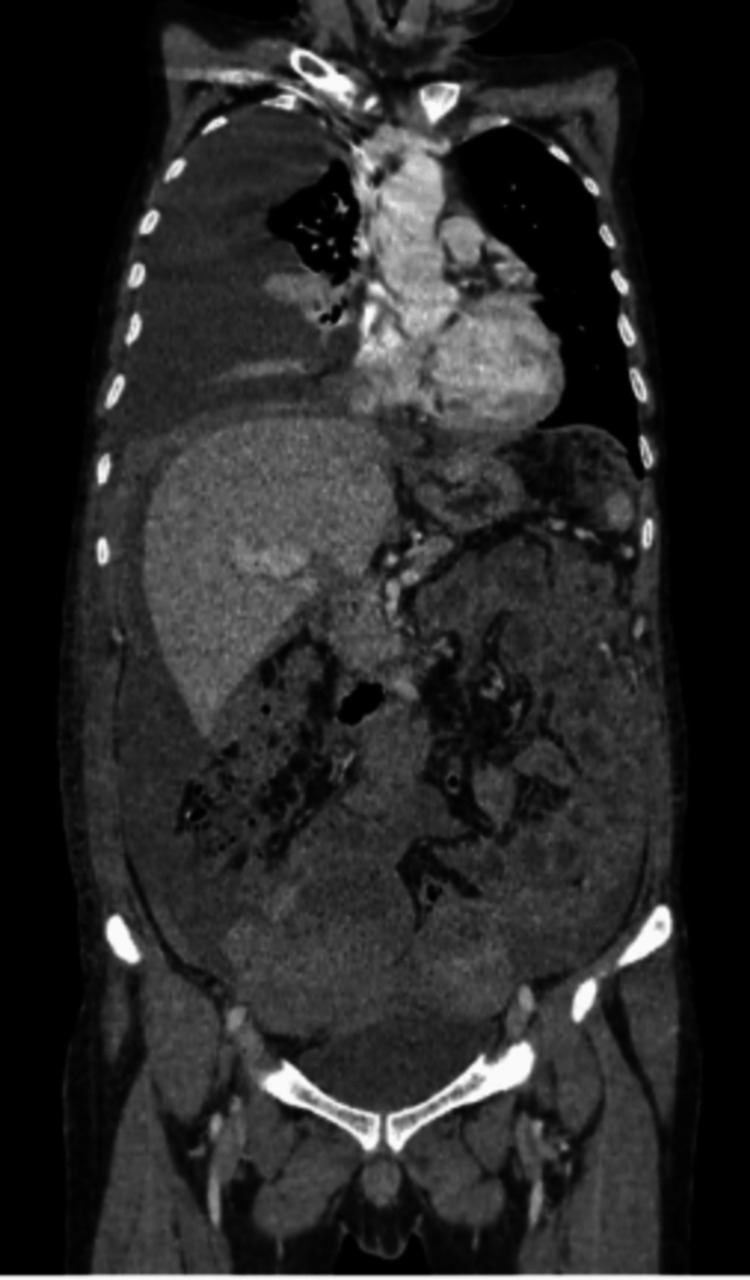
Coronal image of a CT scan showing around 15×15 cm mass in the pelvis CT: computed tomography

CT CAP revealed a 15 cm pelvic mass with extensive pelvic, retroperitoneal, and subcarinal lymphadenopathy, accompanied by peritoneal carcinomatosis, large-volume ascites, and right-sided pleural effusion (Figures 6, 7). The histopathological examination of a biopsy obtained from the pelvic mass confirmed the diagnosis of seminoma.

The patient was urgently admitted for the management of suspected tumor lysis syndrome, supported by laboratory findings (Table 5).

**Table 5:** Investigations pre- and post-rasburicase and pre- and post-allopurinol AFP, alpha-fetoprotein; K, potassium; LDH, lactate dehydrogenase; HCG, human chorionic gonadotropin; PO4, phosphate

	Reference range	Bloods on presentation (14/10/22)	Bloods on 16/10/22 after rasburicase and allopurinol	One week post-rasburicase
Uric acid	3.7-7.7 mg/dL	20	1.1	4.2
K	3.5-5.1 mmol/dL	4.06	3.12	4.58
PO_4_	2.9-4.7 mg/dL	5.6	3.9	3.87
Calcium	8.5-10.5 mg/dL	9.2	9	9.1
Creatinine	0.90-1.30	4.5	3.46	0.98
AFP	<2 IU/L	2.7	-	-
LDH	135-225 U/L	4418	-	-
Beta-HCG	<2 ng/mL	122	-	-

The patient was initiated on intravenous hydration, rasburicase, and allopurinol for the management of tumor lysis syndrome. Following improvement in renal function, chemotherapy was commenced with etoposide at a 25% dose reduction, full-dose bleomycin, and carboplatin substituted for cisplatin due to impaired renal function. Subsequently, he tolerated three additional cycles of the standard BEP regimen without significant complications.

Posttreatment CT imaging demonstrated a reduction of the right scrotal mass to 35 mm and the pelvic mass to 55 mm. The patient was placed on routine surveillance with CT scans every three months. One year after the completion of chemotherapy, the most recent imaging showed further regression, with the right scrotal mass measuring 26 mm and the pelvic mass 42 mm, consistent with ongoing disease control.

Case 6

**Figure 8:**
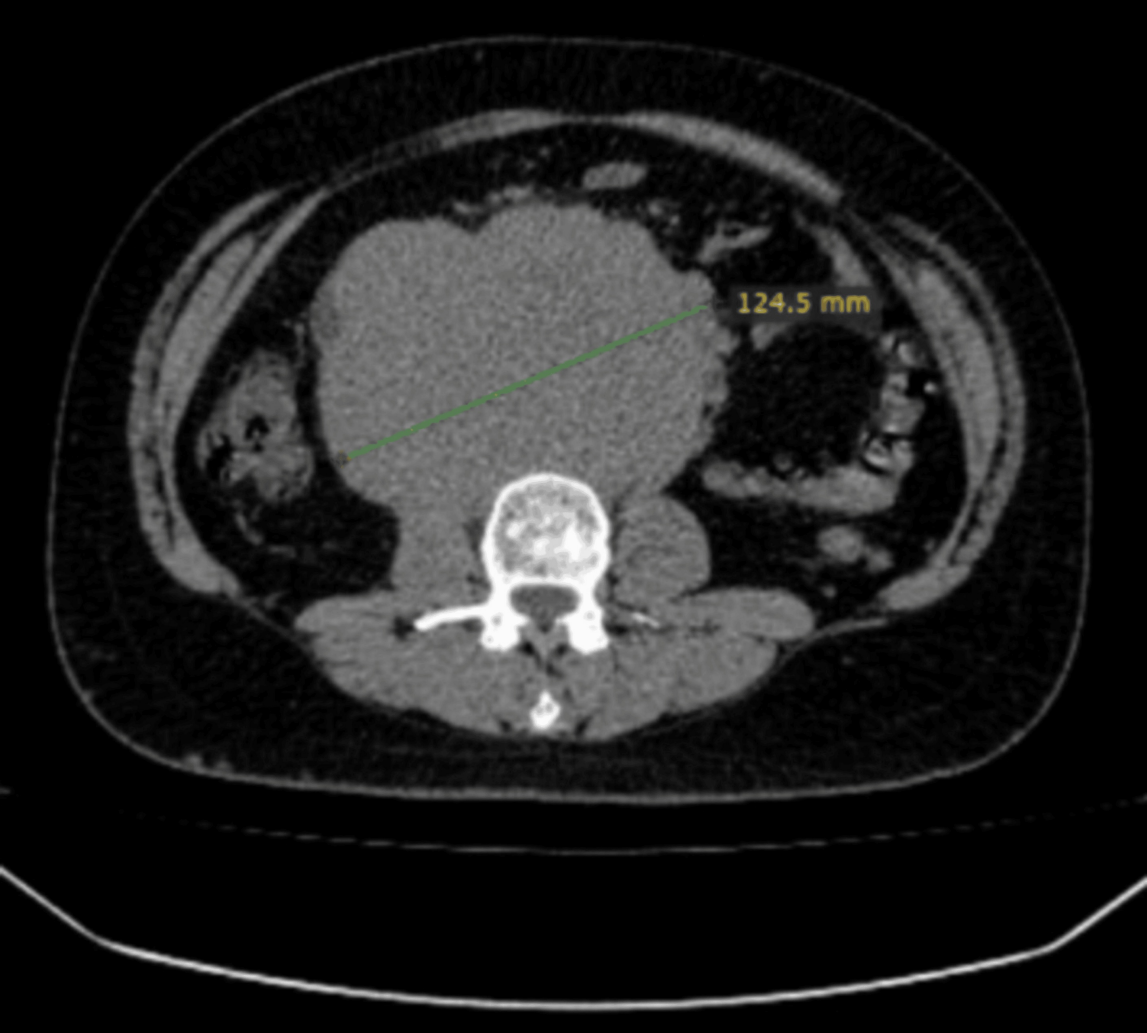
Axial image of a CT scan showing a mass of 11 cm in the abdomen CT: computed tomography

**Figure 9:**
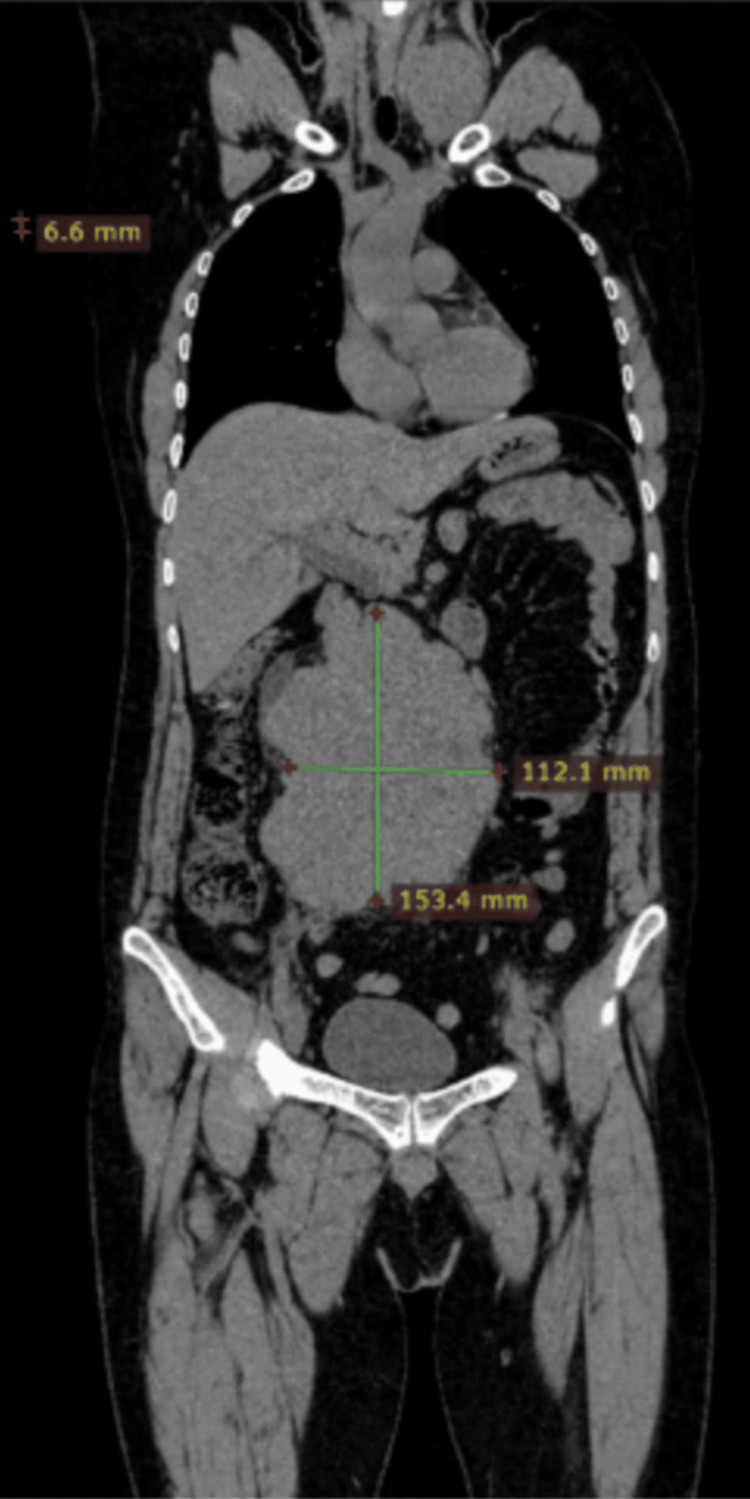
Coronal image of a CT scan showing a mass of 11 cm in the abdomen CT: computed tomography

A 34-year-old gentleman with a history of undescended testes presented with a one-month history of abdominal pain and vomiting. Physical examination revealed abdominal distension and atrophic, small-sized testes. A contrast-enhanced CT of the chest, abdomen, and pelvis demonstrated an 11×8 cm retroperitoneal mass, left cervical lymphadenopathy, and moderate right-sided hydronephroureter (Figures 8, 9). A biopsy of the cervical lymph node confirmed metastatic seminoma. Tumor markers showed elevated beta-HCG at 73 ng/mL (reference range: <2 ng/mL), LDH at 1712 U/L (reference range: 135-225 U/L), and normal AFP levels. Renal function tests revealed a creatinine of 11 mg/dL (reference range: 0.90-1.30), potassium of 5.6 mmol/L (reference range: 3.5-5.1), calcium of 8.7 mg/dL (reference range: 8.5-10.5), uric acid of 15 mg/dL (reference range: 3.7-7.7), and phosphate of 6.7 mg/dL (reference range: 2.9-4.7). Complete blood count was within normal limits.

Given the evidence of spontaneous tumor lysis syndrome and obstructive uropathy, the patient was started on rasburicase, and bilateral nephrostomy tubes were inserted. Following the normalization of renal function, chemotherapy was initiated with cisplatin at 50% dose reduction and full-dose etoposide. The patient was subsequently discharged.

Two days post-chemotherapy, he presented emergently with diarrhea, vomiting, and neutropenic sepsis. He required intensive care unit admission for fluid-refractory hypotension and multiorgan dysfunction. Despite ventilatory support and triple vasopressor therapy, the patient’s condition deteriorated, and he succumbed within 24 hours.

## Discussion

Tumor lysis syndrome (TLS) is an uncommon complication in testicular germ cell tumors (TGCTs), with spontaneous TLS reported infrequently in the literature [5-8,10-13]. While acute TLS typically occurs following cytotoxic chemotherapy, other precipitating factors include radiotherapy, corticosteroids, and tyrosine kinase inhibitors [2,3,9]. The laboratory diagnostic criteria for TLS require the presence of two or more abnormal serum values among uric acid, potassium, phosphate, or calcium, with a ≥25% change from baseline considered significant [5,8,14,15].

This case series highlights our clinical experience with the presentation, diagnosis, and management of spontaneous TLS in TGCT, representing, to our knowledge, the largest of such series reported to date. All patients exhibited a high tumor burden on imaging and were promptly diagnosed based on laboratory findings [13,14]. Despite timely intervention, mortality remains substantial and is consistent with outcomes reported in prior literature [7,14-17].

Given the rarity of TLS in TGCT, it is crucial to exclude other causes of acute kidney injury (AKI), including prerenal, intrinsic renal, and post-renal etiologies [8,9]. Multifactorial AKI was observed in several cases within our series, which aligns with previous descriptions of electrolyte abnormalities, uric acid nephropathy, obstructive uropathy, and sepsis contributing to renal impairment in TLS [8,12,14]. Hemodialysis proved effective in correcting metabolic derangements and managing fluid overload [8,12,17]. Impaired renal function often precludes the administration of standard cisplatin-based chemotherapy in the initial cycle; thus, we employed carboplatin and renal dose-adjusted etoposide and bleomycin as a bridging regimen, consistent with accepted oncology practice [6,18,19].

Interestingly, the majority of patients in our series had seminoma, either biopsy-confirmed or inferred biochemically, suggesting a potential predilection for spontaneous TLS in seminomatous tumors [6,13]. Mortality associated with spontaneous TLS remains high, reported up to 50%, primarily due to renal failure, sepsis, and cardiac arrhythmias [7,8,14,17]. These patients warrant early multidisciplinary involvement, including intensive care and nephrology teams, to optimize monitoring and management [8,9,12,17-19].

Limitations

This study has several limitations inherent to small case series. First, the sample size was limited to six patients from a single center, which restricts the generalizability of our findings and precludes robust statistical analysis. Second, the retrospective nature of the study may introduce selection and information bias, as data were dependent on the accuracy and completeness of medical records. Third, histological confirmation was not available for all cases, and in some patients, the diagnosis of seminoma was inferred from biochemical and clinical features rather than tissue pathology. Additionally, the heterogeneity in disease burden, comorbid conditions, and management strategies may have influenced clinical outcomes, making it difficult to draw definitive conclusions regarding optimal treatment approaches. Finally, given the rarity of spontaneous tumor lysis syndrome in testicular germ cell tumors, our observations should be interpreted as hypothesis-generating, and larger multicenter studies are required to better characterize the risk factors, optimal management, and outcomes of this condition.

## Conclusions

Spontaneous tumor lysis syndrome is a rare but catastrophic complication of testicular germ cell tumors that can occur prior to the initiation of cytotoxic therapy, particularly in patients with extensive tumor burden. This case series highlights that even seminomatous disease, typically considered indolent, may be associated with severe metabolic derangements and high mortality when complicated by spontaneous TLS. Early recognition, the prompt initiation of aggressive supportive measures, and close multidisciplinary collaboration are essential to mitigate end-organ damage. Furthermore, the careful individualization of chemotherapy timing and dosing is critical to balance oncologic efficacy with metabolic safety. Increased clinical awareness and early risk stratification may improve outcomes in this otherwise highly curable malignancy.
